# Phylogenetic analysis of faecal microbiota from captive cheetahs reveals underrepresentation of Bacteroidetes and *Bifidobacteriaceae*

**DOI:** 10.1186/1471-2180-14-43

**Published:** 2014-02-18

**Authors:** Anne AMJ Becker, Myriam Hesta, Joke Hollants, Geert PJ Janssens, Geert Huys

**Affiliations:** 1 Laboratory of Microbiology, Department of Biochemistry and Microbiology, Faculty of Sciences, Ghent University, Ghent, Belgium; 2 Laboratory of Animal Nutrition, Department of Nutrition, Genetics and Ethology, Faculty of Veterinary Medicine, Ghent University, Merelbeke, Belgium; 3 BCCM/LMG Bacteria Collection, Faculty of Sciences, Ghent University, Ghent, Belgium

**Keywords:** Bacterial community sequencing, Exotic felids, Wildlife conservation, Zoo nutrition

## Abstract

**Background:**

Imbalanced feeding regimes may initiate gastrointestinal and metabolic diseases in endangered felids kept in captivity such as cheetahs. Given the crucial role of the host’s intestinal microbiota in feed fermentation and health maintenance, a better understanding of the cheetah’s intestinal ecosystem is essential for improvement of current feeding strategies. We determined the phylogenetic diversity of the faecal microbiota of the only two cheetahs housed in an EAZA associated zoo in Flanders, Belgium, to gain first insights in the relative distribution, identity and potential role of the major community members.

**Results:**

Taxonomic analysis of 16S rRNA gene clone libraries (702 clones) revealed a microbiota dominated by Firmicutes (94.7%), followed by a minority of Actinobacteria (4.3%), Proteobacteria (0.4%) and Fusobacteria (0.6%). In the Firmicutes, the majority of the phylotypes within the Clostridiales were assigned to *Clostridium* clusters XIVa (43%), XI (38%) and I (13%). Members of the Bacteroidetes phylum and *Bifidobacteriaceae*, two groups that can positively contribute in maintaining intestinal homeostasis, were absent in the clone libraries and detected in only marginal to low levels in real-time PCR analyses.

**Conclusions:**

This marked underrepresentation is in contrast to data previously reported in domestic cats where Bacteroidetes and *Bifidobacteriaceae* are common residents of the faecal microbiota. Next to methodological differences, these findings may also reflect the apparent differences in dietary habits of both felid species. Thus, our results question the role of the domestic cat as the best available model for nutritional intervention studies in endangered exotic felids.

## Background

In the broad scope of wildlife conservation with the aim to protect animal species from extinction, researchers and zoo managers face significant challenges in the conservation of threatened and endangered species. In zoo animal husbandry, nutrition is one of the most critical components [1]. Feeding mismanagement may give rise to suboptimal health, low breeding performance and a higher incidence of gastrointestinal and metabolic diseases [2-4]. In this context, well-balanced diets represent an important route for prevention or therapeutic intervention [5,6].

Due to diet-induced evolutionary adaptations, cats have developed a strictly carnivorous lifestyle with unique nutrient requirements [7]. Extrapolations of the dietary profile of the domestic cat to wild felids in captivity have been made [8,9] but are highly debatable since great differences exist in regards to their anatomical, behavioral and nutritional characteristics. Domestic cats are subjected to frequent feeding portions of carbohydrate-rich extruded kibble diets [10]. In contrast, captive exotic felids are usually fed once a day a commercially prepared raw meat diet, sometimes supplemented with a vitamin and mineral premix, or whole carcasses [11]. The latter comes with variable amounts of indigestible animal tissues, such as raw bones, tendons, cartilage, skin, hair or feather. The undigested portion of the diet provides the main source of fermentable substrates for the intestinal microbiota, which form the main go-between in the translation of nutritional properties of the diet to health benefits for the host [12]. Comparison of mammalian gut microbiotas has shown that diet is, next to gut physiology, a major regulator of faecal microbiota composition [13].

In domestic cats, taxonomic and functional studies of the intestinal microbial communities have shown that different sources of dietary fibre (*i.e.*, cellulose, pectin, fructooligosaccharide) modified the composition of bacterial phyla in the faeces. For instance, cats fed a diet containing 4% pectin were found to display a higher percentage of Firmicutes and Spirochaetes than cats fed a diet containing 4% cellulose [14]. In the same study, dietary fructooligosaccharides increased the percentage of Actinobacteria. Conversely, high-protein diets induced a microbial shift towards decreased *E. coli*, *Bifidobacterium* and *Lactobacillus* populations [15,16]. In captive exotic felids, however, information on the composition and dietary modulation of the intestinal microbiota remains scarce [8].

Recent *in vivo* and *in vitro* studies in one of the most endangered exotic felid species, the cheetah (*Acinonyx jubatus)*, point towards a significant role for microbial degradation of undigested animal tissues in the host’s metabolic homeostasis [17,18]. However, because the number of captive animals available for well-documented faecal sample collection is extremely limited and because the composition and the functional capacity of the cheetah microbiota is virtually unknown, it has not been possible to link these observations to specific bacterial shifts or adaptations in the intestinal ecosystem. In addition, direct extrapolation of microbiological insights obtained for the domestic cat is not a valid approach given its adaptation to commercial diets. To start bridging the knowledge gap between the design of nutritional intervention strategies and the prediction of potential health benefits, this study aimed to inventorize the predominant faecal microbiota of the only two captive cheetahs held in a zoo in Flanders (Belgium) associated with the European Association of Zoos and Aquaria (EAZA). Compositional analysis of 16S rRNA gene clone libraries was used for classification of the obtained phylotypes at phylum and family level, leading to the identification of the major bacterial groups that compose the cheetah’s intestinal ecosystem.

## Methods

### Sample collection

Fresh faecal samples (200 gram) were collected in 2011 from the two adult male cheetahs (B1 and B2; both 10 years old) housed at Zooparc Planckendael (Flanders, Belgium), a full member of EAZA (http://www.eaza.net/membership). The animals shared indoor and outdoor housing and were fed their regular zoo diet *i.e.* chunked boneless horsemeat (2 kg/day/animal) topdressed with a vitamin and mineral premix (Carnicon®; Aveve, Leuven, Belgium) randomly interspersed with unsupplemented whole rabbits. No medical or health problems were reported or apparent on remote examination, and both cheetahs were treated prophylactically for internal parasites (Horseminth®; Pfizer, Brussels, Belgium). Faecal samples were immediately collected upon defaecation into plastic tubes, transported on dry ice and stored at −80°C until further analysis.

### DNA extraction

Prior to DNA extraction, 25 grams (wet weight) of each thawed faecal sample was placed separately in sterile stomacher bags and homogenized in 225 ml peptone-buffered saline (PBS) (0.1% [wt/vol] bacteriological peptone [L37; Oxoid, Basingstoke, United Kingdom], 0.85% [wt/vol] NaCl [106404; Merck, Darmstadt, Germany]). The sludgy homogenate was filtered on a Büchner funnel to discard large particles such as hair and bones, and subsequently divided into 1.5 ml aliquots which were stored at −80°C.

The protocol of Pitcher et al. [19] was used in a modified version [20] to extract total bacterial DNA from the faecal samples. DNA size and integrity were assessed on 1% agarose electrophoresis gels stained with ethidium bromide. DNA concentration and purity were determined by spectrophotometric measurement at 234, 260 and 280 nm. DNA extracts were finally diluted ten times with TE buffer (1 mM EDTA [324503; Merck, Darmstadt, Germany], 10 mM Tris–HCl [648317; Merck, Darmstadt, Germany]) and stored at −20°C.

### Real-time PCR

Quantitative PCR amplification and detection were performed using the Roche Light Cycler 480 machine with the Roche Light Cycler 480 SYBR Green I Master kit. Each PCR reaction included 40 ng DNA. Specific primers were used for Bacteroidetes (Bact934F [5′ GGARCATGTGGTTTAATTCGATGAT 3′] and Bact1060R [5′ AGCTGACGACAACCATGCAG 3′]) and Firmicutes (Firm934F [5′ GGAGYATGTGGTTTAATTCGAAGCA 3′] and Firm 1060R [5′ AGCTGACGACAACCATGCAC 3′]), along with universal primers for total bacteria (Eub338F [5′ ACTCCTACGGGAGGCAGCAG 3′] and Eub518R [5′ ATTACCGCGGCTGCTGG 3′]) as previously described [21]. Samples were incubated at 95°C for 5 min and subsequently amplified during 45 cycles of 95°C for 10 s, 60°C for 30 s, and 72°C for 1 s. The relative amount of Firmicutes and Bacteroidetes 16S rRNA in each sample was normalized to the total amount of faecal bacteria amplified with 16S rRNA gene-based universal primers [22,23]. *Bifidobacteriaceae* were quantified using *Bifidobacterium*-specific primers g-Bifid-F (5′ CTCCTGGAAACGGGTGG 3′) and g-Bifid-R (5′ GGTGTTCTTCCCGATATCTACA 3′) [24].

The ability of primers Bact934F and Bact1060R to detect members of the Bacteroidetes phylum in cheetah faeces was evaluated in a spiking experiment. For that purpose, *Bacteroides fragilis* DSM 1396, *Bacteroides uniformis* DSM 6597 and *Bacteroides distansonius* DSM 20701 were cultured anaerobically at 37°C for 48 h on Reinforced Clostridial Medium (RCM) (M37; Oxoid, Basingstoke, United Kingdom). Inocula were prepared from harvested colonies and enumerated by plating serial 10-fold dilutions. Similarly, RCM counts were determined for faecal homogenates of B1 and B2. These homogenates were spiked with an equivalent mixture of the three *Bacteroides* strains at 1%, 10% and 50% of the total RCM count. Spiked samples were subjected to DNA-extraction and real-time PCR as described above.

### Community PCR

Template DNA obtained from cheetahs B1 and B2 was subjected to 16S rRNA gene amplification using the conserved primers pA (5′ AGA GTT TGA TCC TGG CTC AG 3′) and pH (5′ AAG GAG GTG ATC CAG CCG CA 3′) which flank respectively the extreme 5′ and 3′ part of the 16S rRNA gene, thus allowing amplification of the entire gene [25]. Each reaction mixture (50 μl) contained 5 μl 10x PCR buffer (100 mM Tris–HCl, pH 8.3 [at 25°C]; 500 mM KCl; 15 mM MgCl_2_; 0.01% [wt/vol] gelatin [GeneAmp®; Applied Biosystems, USA]), 1 μl 25 mM MgCl_2_, 5 μl 2 mM dNTPs (GeneAmp®; Applied Biosystems, USA), 0.04 μl 10 μg/μl bovine serum albumin, 1.25 μl 1 U/μl AmpliTaq® (Applied Biosystems, USA), 2.5 μl of each 10 μM primer, 4 μl template DNA and milliQ water to 50 μl. The samples were amplified in the Veriti™ Dx 96-Well Thermal Cycler (Applied Biosystems, USA), using the following PCR programme: initial denaturation at 94°C for 5 min followed by 18 cycles of 94°C for 1 min, 55°C for 1 min and 72°C for 1 min, with a final extension of 72°C for 10 min. Negative (milliQ water as template) and positive controls (*Marinobacter* sp. strain T278 [R-39409]) were included in parallel. Amplicons were checked on a 1% agarose gel under UV illumination after ethidium bromide staining of the gel, and subsequently purified with the QIAquick® PCR purification kit (Qiagen, Germany).

### Cloning of bacterial 16S rRNA gene amplicons

For both cheetahs B1 and B2, a clone library was prepared. Purified 16S rRNA gene amplicons were ligated into the pGEM®-T Vector System (Promega Benelux, The Netherlands) and transformed into competent *E. coli* cells according to the manufacturer’s instructions. White clones were amplified using the primer pair T7 (5′ AAT ACG ACT CAC TAT AGG 3′) and Sp6 (5′ ATT TAG GTG ACA CTA TAG 3′) to determine the size of the inserts.

### Sequencing and sequence processing

The diversity of the clone libraries was examined via short fragment sequencing on an ABI PRISM 3130xl Genetic Analyzer (Applied Biosystems, USA) by means of the Big Dye® XTerminator™ v.3.1. Cycle Sequencing and Purification Kit (Applied Biosystems, USA) according to the protocol of the supplier. The sequencing primer used was BKL1 [26]. For each sample, clones were sequenced, assembled in BioNumerics (Applied Maths, Sint-Martens-Latem, Belgium) and edited to exclude the primer binding sites. Chimeras were detected using Bellerophon [27] and B2C2 [28], and excluded for further analysis.

### Phylogenetic analyses

Chimera-free sequences were aligned using ClustalW in MEGA 5.0 [29] and corrected by manual inspection. Homology searches were performed via BLAST [30], and taxonomic classification of the 16S rRNA transcripts was obtained by comparison against The Ribosomal Database Project-II (RDP) [31]. Only annotations with a bootstrap value over 0.8 were considered as well identified phylogenetic levels, leaving successive levels as unclassified. Groups of sequences with ≤ 3% sequence divergence (≥ 97% similarity) were defined as an operational taxonomic unit (OTU) or phylotype. Rarefaction curves were determined for different clone library sizes and Good’s coverage index [32] was calculated as 1-(n/N) × 100, where n is the number of singleton phylotypes and N is the total number of sequences in the sample. From each OTU at the 97% cut off, a representative clone was selected along with its nearest type strain from the RDP database. A similarity-matrix was calculated using the Maximum Composite Likelihood parameter and data were visualized in a neighbour-joining phylogenetic tree constructed in MEGA 5.0. Reliability of the tree was evaluated based on 1000 bootstrap replicates.

### Availability of supporting data

The data set supporting the results of this article is available in the GenBank repository, accession numbers KF909375 – KF910074, and the phylogenetic tree has been deposited at TreeBase (http://treebase.org/treebase-web/search/study/trees.html?id=15139).

## Results

### Distribution of OTUs in 16S rRNA gene clone libraries

Two clone libraries (CL-B1 and CL-B2) were created using the full-length 16S rRNA gene amplicons from samples B1 and B2. Although most of the DNA inserts corresponded to the expected full-length amplification products, some clones contained short fragments probably due to internal restriction sites. A selection of 384 clones per library was sequenced with primer BKL1, resulting in 352 and 350 quality-checked sequences of 400 to 450 bp length from the 5′ end for libraries CL-B1 and CL-B2, respectively. With a 97% sequence identity criterion, 29 OTUs were obtained for CL-B1 and 37 OTUs for CL-B2. The coverage of the clone libraries was 98.6% and 97.7%, respectively, according to Good’s formula [32]. Among the 66 OTUs, only 18 were found to be common to both libraries. Together, these common OTUs represented 298 sequences (84.7%) in CL-B1 and 317 sequences (90.6%) in CL-B2. Among the remaining OTUs, 11 OTUs were unique to clone library B1 and 19 to clone library B2. Rarefaction curves were obtained by plotting the number of phylotypes observed from both samples against the number of clones sequenced. The decrease in the rate of phylotype detection indicates that the majority of the predominant bacterial diversity in these samples was covered by clone library analysis [see Additional file 1].

### Taxonomic composition of 16S rRNA gene clone libraries at phylum and family level

Firmicutes was by far the most abundant bacterial phylum representing 96.6% and 92.9% of all sequences in CL-B1 and CL-B2, respectively. Three other bacterial phyla formed a minority in the phylogenetic spectrum, *i.e.* Actinobacteria (3.1% in CL-B1; 5.4% in CL-B2), Proteobacteria (0.3% in CL-B1; 0.6% in CL-B2) and Fusobacteria (1.1% in CL-B2). Surprisingly, none of the sequences was assigned to the Bacteroidetes phylum, a group of gram-negative bacteria that make up a major part of the mammalian distal intestinal microbiota [33]. To validate the results obtained by sequencing, we determined the relative concentrations of Firmicutes and Bacteroidetes with real-time PCR. The Firmicutes/Bacteroidetes ratio for faecal samples of B1 and B2 was 1/0.0004 and 1/0.0081, respectively, indicating a very low abundance of Bacteroidetes. In spiked faecal samples, however, *Bacteroides* spp. were succesfully recovered down to 1% (10^4^ CFU/ml).

**Figure 1:**
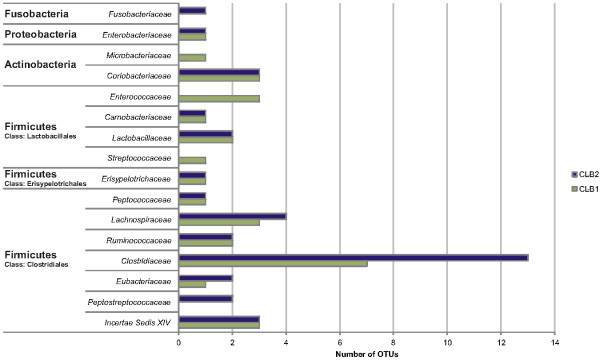
Phylotype frequency at the family level as revealed by clone library analysis of captive cheetah faeces.

Taxonomic assignment at family level revealed 16 different families of which *Clostridiaceae*, *Ruminococcaceae*, *Peptococcaceae* and the unclassified Clostridiales Incertae Sedis XIV held most representatives. Of all these families, the *Clostridiaceae* represented by far the highest number of different phylotypes (Figure 1). The distribution of common OTUs within the predominant bacterial families confirms the phylotype richness of *Clostridiaceae* in both libraries (Table 1).

**Table 1:** Most abundant OTUs, their taxonomic assignment at family level and closest type strain in number and % of clones for both clone libraries from captive cheetah faeces

**OTU**^**a**^	**Bacterial family**	***Clostridium *cluster**	**Closest type strain**	**CL-B1 (352 clones)**	**CL-B2 (350 clones)**
**OTU-2**	***Clostridiaceae***	I	*Clostridium perfringens* ATCC 13124^T^	6 (1.7%)	59 (16.9%)
**OTU-3**	***Clostridiaceae***	XI	*Clostridium hiranonis* TO-931^T^	48 (13.6%)	138 (39.4%)
**OTU-5**	***Clostridiaceae***	XI	*Clostridium glycolicum* DSM 1288^T^	1 (0.3%)	14 (4.0%)
**OTU-6**	***Peptococcaceae***	n/a	*Desulfonispora thiosulfatigenes* DSM 11270^T^	33 (9.4%)	1 (0.3%)
**OTU-7**	***Ruminococcaceae***	XIVa	*Ruminococcus gnavus* ATCC 29149^T^	69 (19.6%)	20 (5.7%)
**OTU-10**	**Incertae Sedis XIV**	XIVa	*Blautia hansenii* JCM 14655^T^	36 (10.2%)	19 (5.4%)
**OTU-12**	**Incertae Sedis XIV**	XIVa	*Blautia glucerasei* HFTH-1^T^	32 (9.1%)	3 (0.9%)
**OTU-13**	**Incertae Sedis XIV**	XIVa	*Blautia glucerasei* HFTH-1^T^	29 (8.2%)	8 (2.3%)
**OTU-17**	***Coriobacteriaceae***	n/a	*Collinsella stercoris* RCA55-54^T^	6 (1.7%)	13 (3.7%)
**OTU-25**	***Enterococcaceae***	n/a	*Enterococcus cecorum* ATCC 43198^T^	31 (8.8%)	-

^a^OTUs which consist of at least ≥ 10 clones in CL-B1 or CL-B2; OTU = operational taxonomic unit; n/a = not applicable.

### Phylogenetic analysis of 16S rRNA gene clone libraries at OTU level

**Figure 2:**
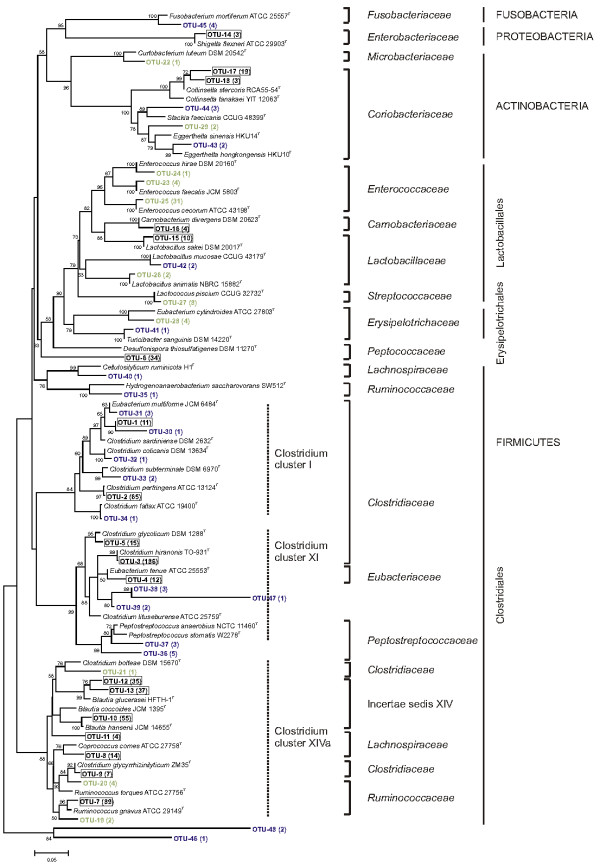
**Neighbour-joining phylogenetic tree showing the nearest phylogenetic related type strains for recovered OTUs from two 16S rRNA clone libraries from captive cheetah faeces.** Bootstrap values, expressed as percentages of 1000 replications, above 50% are given at branching points. The scale bar shows 5 nucleotide substitutions per 100 nucleotides. Number of clones in parentheses follows label of either common OTUs (framed), OTUs solely from CL-B1 (green) or CL-B2 (purple).

For each OTU, a representative clone sequence was selected along with the type strain of its nearest validated species neighbour as obtained in RDP to construct a wide-range phylogenetic tree. Figure 2 shows the phylogenetic inferences among the OTUs affiliated with the phyla Firmicutes, Actinobacteria, Proteobacteria and Fusobacteria. Recovered sequences within the Firmicutes spanned three major orders *i.e.* Clostridiales, Lactobacillales and Erysipelotrichales.

Most of the clones fell within the Clostridiales, representing members of seven different bacterial families. A total of 186 clones of this class (31%) belonged to OTU-3 and were highly related (<1% nucleotide divergence) to *Clostridium hiranonis* TO-931^T^. Within the *Clostridiaceae* a high nucleotide similarity was also found for OTU-2, which grouped 65 clones closely to *Clostridium perfringens* ATCC 13124^T^, and for OTU-34, which clustered with *Clostridium fallax* ATCC 19400^T^. However, the latter only consisted of one clone and displayed a low bootstrap value of 56% at its node. For OTU-9, OTU-32 and OTU-5, high bootstrap values (92%, 100% and 95%) and a low nucleotide divergence (1%) indicated their close phylogenetic affiliation to *Clostridium glycyrrhizinilyticum* ZM35^T^, *Clostridium colicanis* DSM 13634^T^ and *Clostridium glycolicum* DSM 1288^T^, respectively. The remaining five OTUs within the *Clostridiaceae* family (OTU-31, OTU-1, OTU-30, OTU-33 and OTU-21) clustered under lower bootstrap values with their respective type strains. The *Ruminococcaceae* family was also well represented by four OTUs of which OTU-7 constituted 89 clones closely related to *Ruminococcus gnavus* ATCC 29149^T^. The high bootstrap value (100%) at the node of cluster OTU-35 and *Hydrogenoanaerobacterium saccharovorans* SW512^T^ suggests a reliable phylogenetic positioning although there was less than 90% sequence similarity between both. The remaining OTU-19 and OTU-20 included only 6 clones clustering at 5% nucleotide divergence with *Ruminococcus gnavus* ATCC 29149^T^ and *Ruminococcus torques* ATCC 27756^T^, respectively. The *Peptococcaceae* family was only represented by OTU-6, which included 34 clones and exhibited a low sequence similarity (80%) with the nearest type strain, *Desulfonispora thiosulfatigenes* DSM 11270^T^. Moreover, the low bootstrap value (63%) questions the phylogenetic position of OTU-6 in this tree. The remaining families *Lachnospiraceae*, *Enterococcaceae* and *Peptostreptococcaceae* were represented by 6 different OTUs which together encompassed 6% of all sequences allocated to the Clostridiales. The unclassified Clostridiales, Incertae Sedis XIV, harbored 18% of all sequences across three OTUs and were all affiliated to the genus *Blautia*. However, only OTU-10 showed 1% sequence divergence to its type strain *Blautia hansenii* JCM 14655^T^, whereas OTU-12 and OTU-13 differed at least 4% from the closest relative *Blautia glucerasei* HFTH-1^T^. Based upon the previously proposed classification of *Clostridium* spp. in phylogenetic clusters [34], Clostridiales sequences from this study fell into three clusters. These included *Clostridium* cluster XIVa (43%), which showed the highest OTU variety containing OTU-7 to OTU-13, *Clostridium* cluster XI (38%) and *Clostridium* cluster I (13%).

Within the Lactobacillales, the bootstrap value of 79% at the node tenuously supports the grouping in four families. Three OTUs together represented by 36 clones grouped in the *Enterococcaceae*. Of these, OTU-24 was closely related to *Enterococcus hirae* DSM 20160^T^ although it only represented one clone with a 3% nucleotide divergence. The other two OTUs (OTU-23 and OTU-25) differed only 1% from the sequences of *Enterococcus faecalis* JCM 5803^T^ and *Enterococcus cecorum* ATCC 43198^T^, respectively. For the *Carnobacteriaceae*, a monophyletic branch at 100% bootstrap support was formed by OTU-16 with *Carnobacterium divergens* DSM 20623^T^. A total of 14 clones all grouping in the *Lactobacillaceae* formed three subclusters, each at 100% bootstrap support with their closest type strain. OTU-15 was phylogenetically linked to *Lactobacillus sakei* DSM 20017^T^, OTU-42 to *Lactobacillus mucosae* CCUG 43179^T^ and OTU-26 to *Lactobacillus animalis* NBRC 15882^T^. Finally, *Streptococcaceae* were represented by OTU-27, which was closely related (1% nucleotide divergence) to *Lactococcus piscium* CCUG 32732^T^.

The order Erysipelotrichales was divided into two distinct clusters representing members of the *Erysipelotrichaceae* family. More specifically, OTU-28 (4 clones) grouped most closely to *Eubacterium cylindroides* ATCC 27803^T^, whereas the single clone of OTU-41 clustered with *Turicibacter sanguinis* MOL 361^T^.

The branching pattern within the phylum Actinobacteria consisted of two families. The *Microbacteriaceae* were represented by a single clone (OTU-22) clustering at 100% bootstrap support with *Curtobacterium luteum* DSM 20542^T^. The *Coriobacteriaceae* comprising the genera *Collinsella*, *Slackia* and *Eggerthella* were represented by five OTUs. Of these, OTU-17 (19 clones) and OTU-18 (3 clones) clustered with *Collinsella stercoris* RCA55-54^T^ and *Collinsella tanakaei* YIT 12063^T^, respectively. The few clones assigned to OTU-29, OTU-43 and OTU-44 were most closely related to *Eggerthella hongkongenis* HKU10^T^, *Eggerthella sinensis* HKU14^T^ and *Slackia faecicanis* CCUG 48399^T^, respectively.

The single OTU belonging to the Proteobacteria, OTU-14 (3 clones), exhibited <2% nucleotide divergence with *Shigella flexneri* ATCC 29903^T^ with 100% bootstrap support. Likewise, the phylum Fusobacteria was only represented by OTU-45 (4 clones), which was phylogenetically most closely related to *Fusobacterium mortiferum* ATCC 25557^T^.

Five OTUs (OTU-38, OTU-39, OTU-46, OTU-47, OTU-48), containing 1 to 3 clones each, failed to clearly group within a particular genus or family. Given that all sequences used for phylogenetic analyses were of good quality, these OTUs may represent species that are currently not included in the RDP database.

### Common diversity of CL-B1 and CL-B2

The faecal community members shared by CL-B1 and CL-B2 encompassed three phyla (Firmicutes, Actinobacteria and Proteobacteria), 10 families and 18 OTUs (OTU-1 to OTU-18). The *Clostridiaceae* family harbored five common OTUs. Of these, OTU-3 (affiliated with *Clostridium hiranonis* TO-931^T^) accounted for 13.6% and 39.4% of all clones in CL-B1 and CL-B2, respectively. Followed by OTU-7 (affiliated with *Ruminococcus gnavus* ATCC 29149^T^) representing 19.6% and 5.7% of all sequences in CL-B1 and CL-B2, respectively (Table 1). On top of the five common OTUs, CL-B2 harbored eight unique OTUs within the family *Clostridiaceae* compared to one unique OTU (OTU-21) for CL-B1. Other shared families within the phylum Firmicutes were the *Peptococcaceae*, *Eubacteriaceae*, *Lachnospiraceae* and unclassified Clostridiales. All of these consisted of common OTUs with the exception of the *Lachnospiraceae* family that also comprised a single clone of OTU-40 in CL-B2. However, the phylogenetic position of OTU-40 displayed 8% nucleotide divergence with the closest type strain, *Cellulosilyticum ruminicola* H1^T^. In the Proteobacteria, only the family *Enterobacteriaceae* was represented with a single common OTU-14 (affiliated with *Shigella flexneri* ATCC 29903^T^), which harbored a minority population of three clones. The phylum Actinobacteria was represented by two common OTUs (OTU-17 and OTU-18) that were phylogenetically related to the *Coriobacteriaceae*.

### Comparison with available 16S rRNA sequences from captive cheetahs

Our dataset of 702 quality-checked sequences was compared with 597 full-length 16S RNA gene sequences retrieved from a large comparative microbiome study of Ley and co-workers [35] in which one faecal sample each of two captive cheetahs from Saint Louis Zoo (St Louis, Missouri, USA) were included. Despite differences in sequence number and sequence length, both datasets were compared with taxonomic RDP annotation. In line with the present study, Bacteroidetes represented only a very marginal share (*i.e.* 1.3%) in Ley et al.’s dataset. At family level, the dominance of *Clostridiaceae* (16.5%) and *Ruminococcaceae* (4.0%) members was also confirmed. The share of *Peptococcaceae* (1.7%) and the unclassified Clostridiales Incertae Sedis (0.8%) in Ley et al.’s dataset was considerably lower compared to our dataset (5% and 18%, respectively). Two other bacterial families, also represented in the dataset of this study, made up a big part of Ley et al.’s dataset, *Peptostreptococcaceae* (13%) and *Lachnospiraceae* (11%).

Taken together, only the *Clostridiaceae*, *Lactobacillacea*e and *Erysipelotrichaceae* families were common to the faecal microbiota of all four cheetahs included in these two studies.

## Discussion

This study set out to determine the predominant faecal microbial communities of captive cheetahs using 16S rRNA gene clone libraries. At the onset of the study, only two animals with well-documented dietary and health records and housed according to EAZA standards were available for this study in Flanders, Belgium. Phylogenetic analysis of the pooled library set revealed a highly complex microbiota covering a broad phylogenetic spectrum. The Firmicutes were by far the most abundant bacterial phylum compared to the minority of Actinobacteria, Fusobacteria and Proteobacteria. Surprisingly, none of the OTUs of both clone libraries were assigned to members of the Bacteroidetes, the phylum that together with the Firmicutes accounts for >98% of the 16S rRNA gene sequences detected in the gut microbiota of vertebrates [13]. The Bacteroidetes comprise important degraders of complex and otherwise indigestible dietary polysaccharides in the large intestine, which leads to the production of short-chain fatty acids that are reabsorbed by the host as energy source [36,37]. Using a variety of methods, Bacteroidetes have been identified as a dominant group in the faecal microbiota of dogs (27-34%) fed experimental diets (30% protein and 20% fat) [38,39], wild wolves (16,9%) feeding on raw meat [40] and grizzly bears (40%) on an omnivorous diet [41]. Feline microbiome studies using 16S rRNA clone libraries or pyrosequencing have also reported that Bacteroidetes is one of the major (0.45%-10%) phyla in the faecal microbiota of cats alongside Firmicutes and Actinobacteria [42,43]. A recent study using 454 pyrosequencing even reported Bacteroidetes to be the most predominant (68%) bacterial phylum in the feline intestinal microbiome [44]. Although relative levels of the dominant phyla in cats seem to vary between studies, likely as a result of differences in methodologies and/or in dietary regimes of the studied cats, one could expect to also find Bacteroidetes in most other felids. The complete absence of Bacteroidetes members in the 16S rRNA clone libraries of the two captive cheetahs contradicts this expectation, but was corroborated by real-time PCR data indicating a hardly detectable concentration of this phylum against a high background of Firmicutes. The finding that *Bacteroides* spp. could be detected in spiked faecal samples at 10^4^ CFU/ml and possibly lower, excludes major detection artefacts introduced during DNA extraction. Further support for our observations are provided by a comparative study of the gut-associated bacterial communities in 60 mammalian species showing that Bacteroidetes is a rare phylum in most carnivores [35]. In that study, 3-15% of the 16S rRNA gene sequences of captive lions, hyenas and bush dogs were phylogenetically linked to Bacteroidetes, whereas only a marginal contribution (<1%) of this phylum was found for captive polar bears and cheetahs. This is comparable to Bacteroidetes levels reported in a recent microbiome study of captive polar bears [45] and our findings for captive cheetahs. The common denominator between the latter two strict carnivores is their protein-rich diet, whereas domestic cats are usually fed commercially prepared diets containing moderate quantities of carbohydrates and plant-derived soluble fibres [46]. This seems to suggest that differences in dietary regimes and feeding habits account for the large variation in Bacteroidetes levels among carnivores. Low proportions of Bacteroidetes have also been reported in giant pandas which belong to the order Carnivora and have a simple intestinal tract, but are feeding on bamboo [47]. Despite their herbivorous lifestyle, studies have shown that the panda faecal microbiota is more similar to other Carnivora than to unrelated herbivores suggesting that next to diet also gut physiology is a regulator of the faecal microbiota composition [13,35].

Within the Firmicutes, the majority of the Clostridiales isolates common to both clone libraries was assigned to *Clostridium* clusters XIVa (43%), XI (38%) and I (13%). Our results are consistent with previous studies that reported a high prevalence of these three *Clostridium* clusters in carnivores [48,49]. Likewise, similar distributions were found in feline microbiome studies using 16S rRNA clone libraries [43,50] or 16S rRNA gene pyrosequencing [42]. Also in the two cheetahs studied by Ley and co-workers [35], similar high abundances of *Clostridium* clusters XIVa and XI were found in two other cheetahs. *Clostridium* cluster XIVa constitutes a major and highly diverse bacterial group in the distal intestines of mammals [51]. This phylogenetically heterogeneous cluster is in both clone libraries represented by *Ruminococcaceae* spp. most closely related to known mucin-degrading organisms such as *Ruminococcus torques* and *Ruminococcus gnavus*[52] as well as members of the recently proposed genus *Blautia*[53]. The latter group comprises important producers of short-chain fatty acids such as butyrate, which is an important source of energy for colonic epithelial cells and has shown to possess anti-inflammatory and anticarcinogenic potential [54,55]. Feline and canine inflammatory bowel diseases have been associated with reduced bacterial species richness and a reduced proportion of *Clostridium* cluster XIVa [56-58]. Noteworthy, the two cheetahs included in our study showed no signs of gastrointestinal disease. *Clostridium* clusters XI and I include saccharolytic fibre-fermenting species but also proteolytic or toxinogenic clostridia [34]. In *Clostridium* cluster XI, 87% of the common sequences displayed >99% sequence similarity to the type strain of *Clostridium hiranonis*. This species was first described in human faeces and displays bile acid 7-α-dehydroxylating activity. In addition, acetic acid and minor amounts of propionic acid and iso-butyric acid are produced from mono- and disaccharides [59]. Ritchie and co-workers [43] found *Clostridium* cluster XI to account for 22% of the faecal microbiota in healthy cats. Up to 86% of the clones assigned to *Clostridium* cluster I in our study were phylogenetically most closely related to the type strain of the potentially pathogenic species *Clostridium perfringens*. However, with reported isolation rates of up to 63% in healthy cats [60], *C. perfringens* should probably be considered as a common commensal of the feline intestine. Moreover, no significant differences in prevalence of either *C. perfringens* or toxigenic *C. perfringens* strains were observed between healthy cats and cats with diarrhea [60].

Protein-rich diets may increase the presence of *Clostridium* cluster I in pet cats and dogs and induce a shift towards a higher prevalence of proteolytic bacterial species [16,61]. A similar dietary influence has also been reported in other carnivores. *Clostridium* cluster I and XI prevailed in polar bears feeding on seals and fish [45] and captive grizzly bears feeding on a regular diet containing up to 31% protein [49]. The latter study indicated that captive grizzly bears consuming a protein-based diet were more prone to carry *C. perfringens* than wild grizzly bears consuming a more plant-based diet. These results suggest a positive correlation between the prevalence of *Clostridium* clusters I and XI and dietary protein content. In the present study, both cheetahs included in our study were fed a protein-rich diet with minimal dietary fibre *i.e.* boneless horsemeat. Therefore, the high proportions of *Clostridium* cluster I and XI in the faecal microbiota of captive cheetahs may be a reflection of their dietary habits.

Common bacterial communities classified in the phylum Actinobacteria harbored solely species belonging to the genus *Collinsella* within the *Coriobacteriaceae*. This family is a frequent resident of the feline gut microbiota [62]. No members were identified of the *Bifidobacteriaceae*, a group of fibre-fermenting gut bacteria that largely contribute to cross-feeding mechanisms leading to the production of butyrate [63,64]. Also in two other studies both using 16S rRNA gene clone libraries to study the faecal microbiota of wild wolves [40] and pet cats [50], no *Bifidobacteriaceae* were encountered. In contrast, other studies have reported the presence of *Bifidobacteriaceae* in the feline faecal microbiota using alternative techniques such as culturing [65], FISH [56] and a chaperonin 60 gene-based clone library [66]. This suggests that differences in methodologies may, at least to some extent, explain the observed differences between studies. In fact, it has been shown that *Bifidobacteriaceae* may be underrepresented in 16S rRNA gene-based studies, possibly due to the use of universal primers that may underestimate the GC-rich Actinobacteria. Therefore, the combined use of universal and genus-specific primers has been suggested to characterize *Bifidobacterium* spp. in intestinal microbiota [43,67,68]. In the present study, real-time PCR enumeration of *Bifidobacterium* revealed a low mean log_10_ number of 4.43 (data not shown). On the one hand, this illustrates the inability of the clone library approach to detect low levels of *Bifidobacterium* in the cheetah faecal samples. On the other hand, the finding of a significantly higher mean log_10_*Bifidobacterium* concentration of 9.13 in faecal samples of five domestic cats with the same real-time PCR protocol (Becker et al., unpublished data) indicates that marked differences exist in bifidobacterial levels of cheetahs and domestic cats. Possibly, these differences reflect the strictly carnivorous diet of captive cheetahs. In fact, *Bifidobacteriaceae* have been negatively correlated with the protein content of the diet [16,69] and only a few studies have reported the presence of bifidobacteria in faeces of carnivores [70].

Finally, the minor share of Fusobacteria and Proteobacteria found in this study is also confirmed in other feline microbiome studies using 16S rRNA gene clone libraries [50] or shotgun sequencing [44]. Felids seem to harbor less Proteobacteria and Fusobacteria compared to other carnivores such as wolves [40] and dogs. In the latter species even, substantial numbers of Fusobacteria have been observed, but the significance of an enriched Fusobacteria population is yet unknown [39]. In the Proteobacteria, a minority of three clones affiliated with *Shigella flexneri* ATCC 29903^T^. This species is principally a primate pathogen causing bacillary dysentery or shigellosis [71]. Cats have not been reported to be naturally infected [72], although these organisms may be transiently excreted in some clinically normal domestic cats [43,44]. The two cheetahs included in this study showed no signs of shigellosis and to our knowledge this type of infection has not been reported in cheetahs thus far.

## Conclusions

This is the first ever study to specifically characterize the predominant faecal bacterial populations of captive cheetahs using a combination of 16S rRNA clone library and real-time PCR analyses. The study revealed a complex microbial diversity predominantly composed of Firmicutes. The abundance of *Clostridium* clusters XIVa, XI and I in this phylum resembles that in the faecal microbiota of other carnivores. However, the near absence of Bacteroidetes and the low abundance of *Bifidobacteriaceae* are in sharp contrast with the situation in domestic cats but in agreement with faecal microbiota composition reported in other Carnivora. In addition to the apparent differences in feeding habbits between both felid species, also our microbiological findings thus question the role of the domestic cat as a suitable model for nutritional intervention studies in captive felids such as cheetahs.

The present study provides a first taxonomic baseline for further characterizations of the diversity and dynamics of the cheetah intestinal ecosystem. To confirm our main findings based on two animals, the collection of fresh and well-documented faecal samples from more captive cheetahs worldwide is the next challenge. Ultimately, the resulting microbial insights may contribute in the optimization of feeding strategies and the improvement of the general health status of cheetahs in captivity.

## Competing interests

The authors declare no conflict of interest.

## Authors’ contributions

GH, GPJJ and MH designed and supervised the study. AAMJB performed sample collection; AAMJB and JH performed clone library and sequence analysis; AAMJB and GH were responsible for the draft and final version of the manuscript. All authors read and approved the final manuscript.

## Supplementary Material

Additional file 1 **Rarefaction curves for bacterial 16S rRNA gene sequences obtained by clone library analysis of captive cheetah faecal samples.** The slopes of corresponding lineair lines indicate a flattening of the rarefaction curves. CL-B1: clone library of faecal samples of captive cheetah B1; CL-B2: clone library of faecal samples of captive cheetah B2. Click here for file
