# Supplementary figures and images for: Phylogenetic analysis of faecal microbiota from captive cheetahs reveals underrepresentation of Bacteroidetes and Bifidobacteriaceae
Source: BMC Microbiol. 2014 Feb 18;14:43. doi: 10.1186/1471-2180-14-43 (PMC3936777; doi:10.1186/1471-2180-14-43)

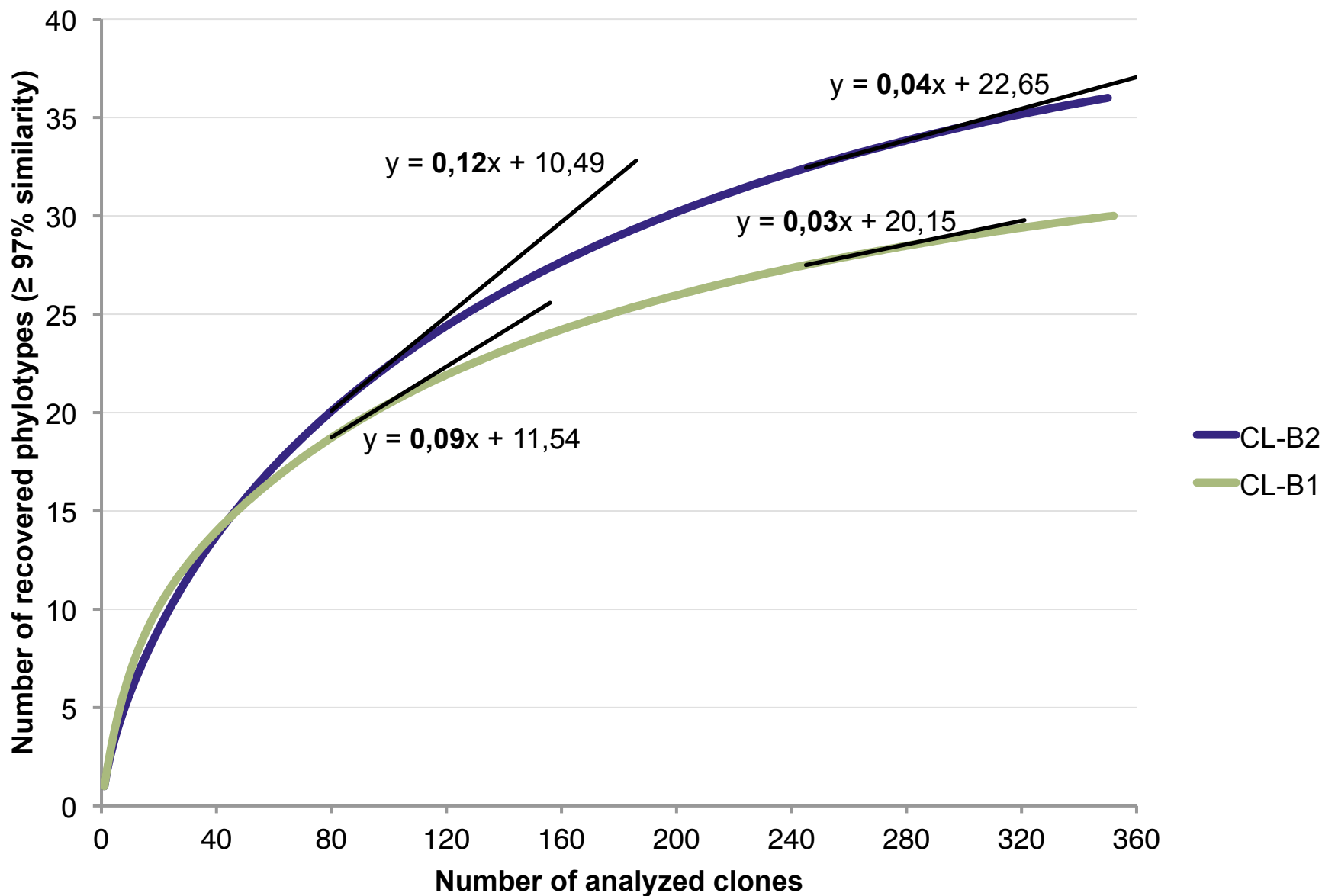

Supplement: Additional file 1 — Rarefaction curves for bacterial 16S rRNA gene sequences obtained by clone library analysis of captive cheetah faecal samples. The slopes of corresponding lineair lines indicate a flattening of the rarefaction curves. CL-B1: clone library of faecal samples of captive cheetah B1; CL-B2: clone library of faecal samples of captive cheetah B2. [file 1471-2180-14-43-S1.pdf]
